# EFNR-WFNR Eastern Europe Regional Meeting in conjunction with the Bistrița Clinical Neuroscience Conference: European Perspectives in Neurorehabilitation

**DOI:** 10.25122/jml-2023-1011

**Published:** 2023-03

**Authors:** Stefana-Andrada Dobran, Alexandra Gherman, Dafin Muresanu

**Affiliations:** 1 RoNeuro Institute for Neurological Research and Diagnostic, Cluj-Napoca, Romania; 2 Sociology Department, Babes-Bolyai University, Cluj-Napoca, Romania; 3 Department of Neuroscience, Iuliu Hatieganu University of Medicine and Pharmacy, Cluj-Napoca, Romania

**Figure 1:**
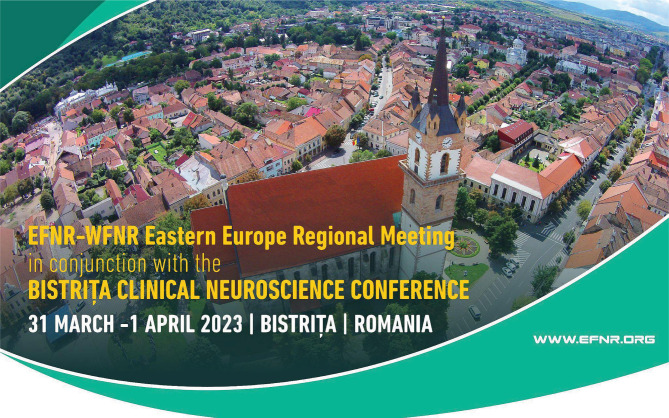
The EFNR-WFNR Eastern Europe Regional Meeting in conjunction with the Bistrița Clinical Neuroscience Conference.

**Figure 2:**
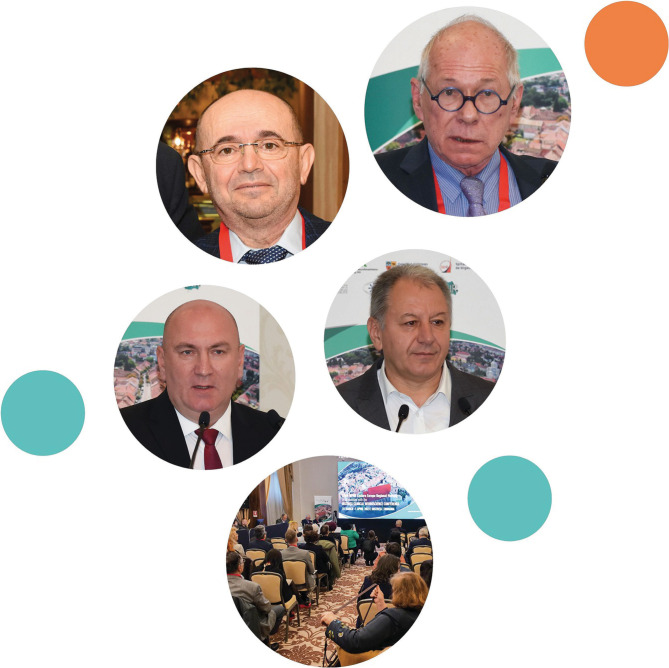
Representatives of the Organizational Committee from the EFNR-WFNR Eastern Europe Regional Meeting in conjunction with the Bistrița Clinical Neuroscience Conference - Prof. Dafin F. Muresanu, EFNR President (upper left), Prof. Volker Homberg, WFNR President (upper right), Gabriel Lazany, Manager of the County Clinical Emergency Hospital Bistrița (middle left), Emil Radu Moldovan, President of the County Council of Bistrița-Năsăud (middle right).

The EFNR-WFNR Eastern Europe Regional Meeting in conjunction with the Bistrița Clinical Neuroscience Conference (Figure 1), took place in Bistrița, Romania, from March 31st to April 1st, 2023. This significant academic gathering was held in a hybrid format and featured esteemed experts from various fields discussing potential advancements in neurorehabilitation. The event was jointly organized by the European Federation of Neurorehabilitation Societies (EFNR), the World Federation for Neurorehabilitation (WFNR), the County Council of Bistrița-Năsăud, and the County Clinical Emergency Hospital Bistrița (Figure 2). It received support from multiple organizations, such as the Foundation of the Society for the Study of Neuroprotection and Neuroplasticity (SSNN), the Foundation for the Study of Nanoneurosciences and Neuroregeneration (FSNN), the Romanian Academy, and the Romanian Society of Neurology (SNR).

The event attracted over 150 participants from Eastern Europe, making it a remarkable success. World-renowned experts gathered to discuss a diverse range of topics, including post-stroke neurorecovery, cognitive decline after stroke, pharmacology in functional recovery, clinical case studies, traumatic brain injury treatment developments, effective stroke recovery, motivation in neurorehabilitation, innovation and sustainability, early rehabilitation after aneurysmal subarachnoid hemorrhage, cerebral veins and dural sinuses thrombosis, autonomic dysfunction in Parkinson's disease, immunomodulatory treatment for multiple sclerosis, complex management of adult SMA patients, neurorehabilitation peculiarities in multiple sclerosis, and computer-assisted and robotic neurorehabilitation for stroke patients. Prof. Dafin F. Muresanu (EFNR President) and Prof. Volker Hömberg (WFNR President) led the scientific event, with experts from around the world sharing their knowledge on various neurorehabilitation subjects.

The WFNR and EFNR are multidisciplinary organizations committed to promoting education, research, and clinical practice in neurorehabilitation. They achieve this through focused initiatives, educational programs, collaborative research, and by enhancing networking opportunities.

Moreover, the Regional Meeting emphasized international viewpoints on current topics while showcasing national experiences and perspectives from countries such as Poland, Serbia, Slovenia, Hungary, the Republic of Moldova, Romania, and Bulgaria. During the March 31st meeting, the current state of neurorehabilitation in Eastern Europe was discussed. This specialized gathering aimed to present the healthcare systems and neurorehabilitation specifics of these countries, focusing on strategies to overcome limitations and improve neurorehabilitation approaches.

The objective of the event was to develop a joint action plan and extend the educational outreach and network of WFNR and EFNR. To accomplish this, the delegations from Poland, Serbia, Bulgaria, Slovenia, the Republic of Moldova, and Romania came together to identify opportunities and challenges in the region. Another goal was to establish institutional partnerships among National Rehabilitation Societies.

A warm welcome address was offered by the town and county representatives along with Prof. Dafin F. Mureșanu, Prof. Volker Hömberg, and Prof. Wolfgang Grisold (World Federation of Neurology President). The experts included Prof. Natan Bornstein (Chairman of the Israeli Stroke Society), Prof. Caterina Pistarini (WFNR Secretary General), Dr. Dana Boering (EFNR Secretary General), Prof. Rodica Bălașa (George Emil Palade University of Medicine, Pharmacy, Sciences and Technology), Prof. László Csiba (Debrecen University), Prof. Iwona Sarzyńska-Długosz (Institute of Psychiatry and Neurology in Warsaw), Prof. Stanislav Groppa (Nicolae Testemițanu State University of Medicine and Pharmacy), Prof. Cătălin Jianu (Victor Babes University of Medicine and Pharmacy), Prof. Ljubica Konstantinovic (University of Belgrade), Prof. Dimitar Maslarov (First University Hospital for Active Treatment St. Joan Krastitel), Prof. Andjela Milovanovic (University of Belgrade), Prof. Cristian Falup-Pecurariu (Transilvania University), Prof. Bogdan O. Popescu (Carol Davila University of Medicine and Pharmacy), Prof. Aleš Pražnikar (Ljubljana University Medical Centre), Prof. Corina Roman (County Hospital Sibiu), Prof. Mihaela Simu (Victor Babeş University of Medicine and Pharmacy Timisoara), Prof. József A. Szász (University of Medicine and Pharmacy Târgu-Mureș), and Prof. Cristina Tiu (Bucharest University Emergency Hospital).

The EFNR-WFNR Eastern Europe Regional Meeting in conjunction with the Bistrița Clinical Neuroscience Conference was a unique event for this region. Hosting such a highly specialized and sophisticated gathering in a relatively small location highlights the growing importance of neurorehabilitation and the global commitment to advancing this field. The successful organization and development of this event in Bistrița demonstrate the potential for growth and collaboration beyond geographical boundaries, challenging traditional ideas as to where top-tier scientific meetings can take place. The Regional Meeting placed Bistrița on an international stage while fostering a sense of community and shared purpose for all attendees, allowing for high-level knowledge exchange.

During the event, Professors Bogdan O. Popescu and Cristina Tiu conducted video interviews with the EFNR team, discussing their views on the Regional Meeting and the National Strategy on Cardiovascular and Cerebrovascular Diseases (SNBCC). Prof. Bogdan O. Popescu emphasized the significance of pharmacological interventions in neurorehabilitation after TBI and the role of multimodal approaches. Prof. Cristina Tiu shared her thoughts on the importance of clinical guidelines for patient management and pharmacological interventions in stroke recovery.

The scientific proceedings concluded with the Bistrita Elites Gala, a special celebration honoring distinguished individuals from science, humanities, and arts to promote development and recognize excellence.

As millions of people worldwide lack access to proper rehabilitation services, and high-tech rehabilitation is often limited, especially in Eastern Europe, innovative and widely accessible solutions are urgently needed. Improved education and coaching strategies for healthcare personnel, community, and caregiver-based approaches, as well as digital communication tools, can help increase access for the general population.

The EFNR-WFNR Eastern Europe Regional Meeting, combined with the Bistrița Clinical Neuroscience Conference, served as a unique forum for knowledge exchange, showcasing Eastern European perspectives on neurorehabilitation and facilitating collaboration among brilliant minds in neurology and neurosciences.

**Figure 3:**
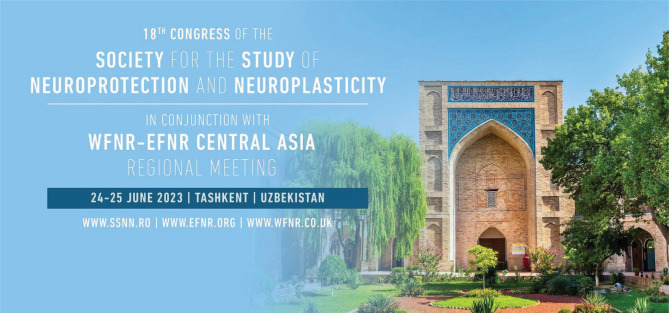
18th Congress of the Society for the Study of Neuroprotection and Neuroplasticity in conjunction with the WFNR-EFNR Central Asia Regional Meeting.

Looking ahead, the WFNR and EFNR will organize a second event, the "18th Congress of the Society for the Study of Neuroprotection and Neuroplasticity, in conjunction with the WFNR-EFNR Central Asia Regional Meeting" (Figure 3). Scheduled from June 24-25 in Tashkent, Uzbekistan, the event will offer online access and focus on the rehabilitation status of Central Asian countries. We encourage specialists worldwide to attend this anticipated congress and contribute their expertise to the development of multidisciplinary neurorehabilitation.

